# Supplementary material for: DeepPheno: Predicting single gene loss-of-function phenotypes using an ontology-aware hierarchical classifier
Source: PLoS Comput Biol. 2020 Nov 18;16(11):e1008453. doi: 10.1371/journal.pcbi.1008453 (PMC7710064; doi:10.1371/journal.pcbi.1008453)
Supplement: S1 Table — (PDF) [file pcbi.1008453.s001.pdf]

Phenotype ID	Phenotype name	$F_{\max}$
HP:0000118	Phenotypic abnormality	0.999 ± 0.000
HP:0000005	Mode of inheritance	0.961 ± 0.005
HP:0010571	Elevated levels of phytanic acid	0.960 ± 0.064
HP:0010965	Abnormal circulating phytanic acid level	0.933 ± 0.107
HP:0010964	Abnormal circulating long-chain fatty-acid concentration	0.893 ± 0.128
HP:0008167	Very long chain fatty acid accumulation	0.893 ± 0.128
HP:0001088	Brushfield spots	0.892 ± 0.130
HP:0000707	Abnormality of the nervous system	0.861 ± 0.008
HP:0012638	Abnormality of nervous system physiology	0.836 ± 0.008
HP:0003572	Low plasma citrulline	0.808 ± 0.154
HP:0003648	Lacticaciduria	0.793 ± 0.168
HP:0000007	Autosomal recessive inheritance	0.778 ± 0.007
HP:0000152	Abnormality of head or neck	0.767 ± 0.008
HP:0000234	Abnormality of the head	0.763 ± 0.010
HP:0000924	Abnormality of the skeletal system	0.762 ± 0.009
HP:0000478	Abnormality of the eye	0.753 ± 0.010
HP:0008316	Abnormal mitochondria in muscle tissue	0.752 ± 0.089
HP:0011842	Abnormality of skeletal morphology	0.750 ± 0.012
HP:0012265	Ciliary dyskinesia	0.749 ± 0.099
HP:0012262	Abnormal ciliary motility	0.749 ± 0.099
HP:0012261	Abnormal respiratory motile cilium physiology	0.749 ± 0.099
HP:0003011	Abnormality of the musculature	0.748 ± 0.007
HP:0012639	Abnormality of nervous system morphology	0.746 ± 0.006
HP:0012759	Neurodevelopmental abnormality	0.733 ± 0.015
HP:0000271	Abnormality of the face	0.722 ± 0.016
HP:0011965	Abnormal circulating citrulline concentration	0.720 ± 0.112
HP:0002011	Morphological abnormality of the central nervous system	0.717 ± 0.006
HP:0011804	Abnormal muscle physiology	0.709 ± 0.014
HP:0025031	Abnormality of the digestive system	0.707 ± 0.015
HP:0032243	Abnormal tissue metabolite concentration	0.705 ± 0.117
HP:0009121	Abnormal axial skeleton morphology	0.698 ± 0.009
HP:0000631	Retinal arterial tortuosity	0.694 ± 0.200
HP:0001939	Abnormality of metabolism/homeostasis	0.693 ± 0.013
HP:0001626	Abnormality of the cardiovascular system	0.689 ± 0.014
HP:0012443	Abnormality of brain morphology	0.686 ± 0.006
HP:0001574	Abnormality of the integument	0.681 ± 0.016
HP:0005181	Premature coronary artery atherosclerosis	0.673 ± 0.181
HP:0001507	Growth abnormality	0.672 ± 0.021
HP:0012373	Abnormal eye physiology	0.669 ± 0.013
HP:0007768	Central retinal vessel vascular tortuosity	0.667 ± 0.167
HP:0003808	Abnormal muscle tone	0.666 ± 0.016
HP:0005522	Pyridoxine-responsive sideroblastic anemia	0.665 ± 0.169
HP:0000119	Abnormality of the genitourinary system	0.663 ± 0.010
HP:0001112	Leber optic atrophy	0.661 ± 0.229
HP:0000576	Centrocecal scotoma	0.660 ± 0.064
HP:0012372	Abnormal eye morphology	0.659 ± 0.013

HP:0010293	Aplasia/Hypoplasia of the uvula	0.657 ± 0.175
HP:0040064	Abnormality of limbs	0.651 ± 0.008
HP:0100022	Abnormality of movement	0.651 ± 0.008
HP:0010979	Abnormality of lipoprotein cholesterol concentration	0.648 ± 0.148
HP:0012758	Neurodevelopmental delay	0.647 ± 0.018
HP:0010980	Hyperlipoproteinemia	0.640 ± 0.154
HP:0000006	Autosomal dominant inheritance	0.640 ± 0.006
HP:0007183	Focal T2 hyperintense basal ganglia lesion	0.634 ± 0.086
HP:0000929	Abnormality of the skull	0.633 ± 0.012
HP:0010469	Absent testis	0.628 ± 0.165
HP:0004360	Abnormality of acid-base homeostasis	0.625 ± 0.047
HP:0000153	Abnormality of the mouth	0.625 ± 0.017
HP:0100547	Abnormality of forebrain morphology	0.624 ± 0.014
HP:0000598	Abnormality of the ear	0.623 ± 0.022
HP:0002060	Abnormality of the cerebrum	0.622 ± 0.011
HP:0012253	Abnormal respiratory epithelium morphology	0.619 ± 0.135
HP:0001941	Acidosis	0.619 ± 0.041
HP:0031816	Abnormal oral morphology	0.617 ± 0.016
HP:0001249	Intellectual disability	0.617 ± 0.012
HP:0000163	Abnormal oral cavity morphology	0.617 ± 0.018
HP:0000512	Abnormal electroretinogram	0.615 ± 0.043
HP:0000951	Abnormality of the skin	0.614 ± 0.018
HP:0008155	Mucopolysacchariduria	0.613 ± 0.251
HP:0002151	Increased serum lactate	0.611 ± 0.029
HP:0005938	Abnormal respiratory motile cilium morphology	0.610 ± 0.142
HP:0012377	Hemianopia	0.610 ± 0.110
HP:0001252	Muscular hypotonia	0.604 ± 0.019
HP:0011442	Abnormality of central motor function	0.602 ± 0.013
HP:0001250	Seizures	0.601 ± 0.016
HP:0002419	Molar tooth sign on MRI	0.601 ± 0.144
HP:0012736	Profound global developmental delay	0.599 ± 0.105
HP:0001263	Global developmental delay	0.596 ± 0.019
HP:0003541	Urinary glycosaminoglycan excretion	0.593 ± 0.275
HP:0002977	Aplasia/Hypoplasia involving the central nervous system	0.593 ± 0.010
HP:0011121	Abnormality of skin morphology	0.590 ± 0.017
HP:0005339	Abnormality of complement system	0.589 ± 0.272
HP:0012103	Abnormality of the mitochondrion	0.589 ± 0.071
HP:0000662	Nyctalopia	0.587 ± 0.055
HP:0002715	Abnormality of the immune system	0.586 ± 0.012
HP:0025032	Abnormality of digestive system physiology	0.585 ± 0.021
HP:0003287	Abnormality of mitochondrial metabolism	0.582 ± 0.066
HP:0031703	Abnormal ear morphology	0.582 ± 0.015
HP:0001972	Macrocytic anemia	0.581 ± 0.103
HP:0011705	First degree atrioventricular block	0.581 ± 0.101
HP:0012334	Extrahepatic cholestasis	0.580 ± 0.336
HP:0003254	Abnormality of DNA repair	0.580 ± 0.256
HP:0012751	Abnormal basal ganglia MRI signal intensity	0.579 ± 0.073

HP:0002490	Increased CSF lactate	0.578 ± 0.063
HP:0030085	Abnormal CSF lactate level	0.578 ± 0.063
HP:0006744	Adrenocortical carcinoma	0.577 ± 0.338
HP:0011844	Abnormal appendicular skeleton morphology	0.572 ± 0.015
HP:0012251	ST segment elevation	0.570 ± 0.102
HP:0031690	Opportunistic infection	0.570 ± 0.264
HP:0011715	Trifascicular block	0.569 ± 0.132

Performance of DeepPheno by phenotypes (Top 100).
