# Supplementary material for: DeepPheno: Predicting single gene loss-of-function phenotypes using an ontology-aware hierarchical classifier
Source: PLoS Comput Biol. 2020 Nov 18;16(11):e1008453. doi: 10.1371/journal.pcbi.1008453 (PMC7710064; doi:10.1371/journal.pcbi.1008453)
Supplement: S2 Table — (PDF) [file pcbi.1008453.s002.pdf]

Phenotype ID	Phenotype name	$F_{\max}$	Number of classes
HP:0000005	Mode of inheritance	0.772 ± 0.005	14
HP:0001263	Global developmental delay	0.579 ± 0.024	5
HP:0012759	Neurodevelopmental abnormality	0.565 ± 0.010	25
HP:0004359	Abnormal circulating fatty-acid concentration	0.553 ± 0.119	7
HP:0004360	Abnormality of acid-base homeostasis	0.540 ± 0.034	12
HP:0001249	Intellectual disability	0.536 ± 0.008	6
HP:0001941	Acidosis	0.526 ± 0.028	6
HP:0012758	Neurodevelopmental delay	0.522 ± 0.015	12
HP:0001252	Muscular hypotonia	0.520 ± 0.019	8
HP:0003808	Abnormal muscle tone	0.516 ± 0.012	27
HP:0010979	Abnormality of lipoprotein cholesterol concentration	0.504 ± 0.154	7
HP:0000556	Retinal dystrophy	0.495 ± 0.039	5
HP:0012253	Abnormal respiratory epithelium morphology	0.491 ± 0.148	5
HP:0012103	Abnormality of the mitochondrion	0.488 ± 0.037	10
HP:0001507	Growth abnormality	0.483 ± 0.014	40
HP:0001250	Seizures	0.482 ± 0.011	22
HP:0000512	Abnormal electroretinogram	0.476 ± 0.057	6
HP:0011804	Abnormal muscle physiology	0.473 ± 0.009	93
HP:0003287	Abnormality of mitochondrial metabolism	0.470 ± 0.024	8
HP:0040195	Decreased head circumference	0.464 ± 0.016	5
HP:0000002	Abnormality of body height	0.461 ± 0.010	15
HP:0030453	Abnormal visual electrophysiology	0.459 ± 0.029	8
HP:0031704	Abnormal ear physiology	0.457 ± 0.016	13
HP:0000240	Abnormality of skull size	0.456 ± 0.014	9
HP:0012547	Abnormal involuntary eye movements	0.455 ± 0.023	6
HP:0001510	Growth delay	0.455 ± 0.013	15
HP:0012345	Abnormal glycosylation	0.455 ± 0.161	5
HP:0004308	Ventricular arrhythmia	0.449 ± 0.069	5
HP:0000364	Hearing abnormality	0.444 ± 0.016	12
HP:0000707	Abnormality of the nervous system	0.444 ± 0.004	649
HP:0000639	Nystagmus	0.443 ± 0.025	5
HP:0000598	Abnormality of the ear	0.439 ± 0.008	67
HP:0100547	Abnormality of forebrain morphology	0.437 ± 0.011	72
HP:0000478	Abnormality of the eye	0.436 ± 0.009	254
HP:0012638	Abnormality of nervous system physiology	0.433 ± 0.006	358
HP:0004322	Short stature	0.432 ± 0.008	10
HP:0003011	Abnormality of the musculature	0.432 ± 0.007	208
HP:0005115	Supraventricular arrhythmia	0.430 ± 0.107	6
HP:0007364	Aplasia/Hypoplasia of the cerebrum	0.428 ± 0.015	13

HP:0012373	Abnormal eye physiology	0.426 ± 0.010	101
HP:0012639	Abnormality of nervous system morphology	0.426 ± 0.004	293
HP:0000496	Abnormality of eye movement	0.425 ± 0.012	32
HP:0002011	Morphological abnormality of the central nervous system	0.423 ± 0.005	229
HP:0002977	Aplasia/Hypoplasia involving the central nervous system	0.422 ± 0.013	28
HP:0000365	Hearing impairment	0.422 ± 0.017	9
HP:0000152	Abnormality of head or neck	0.418 ± 0.007	449
HP:0000118	Phenotypic abnormality	0.416 ± 0.004	3740
HP:0004323	Abnormality of body weight	0.416 ± 0.017	15
HP:0012443	Abnormality of brain morphology	0.412 ± 0.005	150
HP:0002060	Abnormality of the cerebrum	0.410 ± 0.011	64
HP:0100691	Abnormality of the curvature of the cornea	0.410 ± 0.050	5
HP:0000035	Abnormal testis morphology	0.407 ± 0.012	16
HP:0000234	Abnormality of the head	0.406 ± 0.008	435
HP:0000929	Abnormality of the skull	0.406 ± 0.014	92
HP:0040215	Abnormal circulating insulin level	0.403 ± 0.053	5
HP:0009121	Abnormal axial skeleton morphology	0.403 ± 0.011	197
HP:0008669	Abnormal spermatogenesis	0.402 ± 0.025	5
HP:0009116	Aplasia/Hypoplasia involving bones of the skull	0.400 ± 0.023	7
HP:0040214	Abnormal insulin level	0.400 ± 0.060	6
HP:0012372	Abnormal eye morphology	0.396 ± 0.011	164
HP:0100699	Scarring	0.396 ± 0.035	5
HP:0011442	Abnormality of central motor function	0.396 ± 0.009	69
HP:0004325	Decreased body weight	0.395 ± 0.020	8
HP:0001276	Hypertonia	0.395 ± 0.012	17
HP:0002733	Abnormality of the lymph nodes	0.394 ± 0.064	5
HP:0004329	Abnormal posterior eye segment morphology	0.394 ± 0.017	73
HP:0011446	Abnormality of higher mental function	0.393 ± 0.009	39
HP:0000315	Abnormality of the orbital region	0.392 ± 0.021	61
HP:0000517	Abnormality of the lens	0.392 ± 0.021	11
HP:0010442	Polydactyly	0.391 ± 0.076	10
HP:0002650	Scoliosis	0.391 ± 0.019	5
HP:0030791	Abnormal jaw morphology	0.390 ± 0.020	12
HP:0000846	Adrenal insufficiency	0.389 ± 0.056	5
HP:0001637	Abnormal myocardium morphology	0.389 ± 0.012	8
HP:0003119	Abnormal circulating lipid concentration	0.389 ± 0.051	20
HP:0003107	Abnormal circulating cholesterol concentration	0.387 ± 0.027	10

HP:0000119	Abnormality of the genitourinary system	0.387 ± 0.012	294
HP:0001939	Abnormality of metabolism/homeostasis	0.387 ± 0.011	257
HP:0010461	Abnormality of the male genitalia	0.386 ± 0.011	49
HP:0012072	Aciduria	0.385 ± 0.039	10
HP:0000078	Abnormality of the genital system	0.385 ± 0.012	148
HP:0000271	Abnormality of the face	0.384 ± 0.006	336
HP:0025031	Abnormality of the digestive system	0.383 ± 0.014	221
HP:0030956	Abnormality of cardiovascular system electrophysiology	0.383 ± 0.063	38
HP:0011675	Arrhythmia	0.383 ± 0.065	22
HP:0000277	Abnormality of the mandible	0.382 ± 0.019	9
HP:0011821	Abnormality of facial skeleton	0.382 ± 0.015	27
HP:0000153	Abnormality of the mouth	0.381 ± 0.004	150
HP:0000504	Abnormality of vision	0.381 ± 0.011	29
HP:0031703	Abnormal ear morphology	0.381 ± 0.008	60
HP:0001123	Visual field defect	0.381 ± 0.040	7
HP:0000924	Abnormality of the skeletal system	0.381 ± 0.006	631
HP:0025032	Abnormality of digestive system physiology	0.380 ± 0.017	57
HP:0011733	Abnormality of adrenal physiology	0.380 ± 0.031	9
HP:0011443	Abnormality of coordination	0.379 ± 0.025	20
HP:0000811	Abnormal external genitalia	0.378 ± 0.011	62
HP:0001098	Abnormal fundus morphology	0.378 ± 0.016	69
HP:0012243	Abnormal reproductive system morphology	0.377 ± 0.010	119
HP:0100022	Abnormality of movement	0.376 ± 0.010	84
HP:0000366	Abnormality of the nose	0.376 ± 0.008	54

HPO branches with significantly better performance (Top 100)
