# Supplementary material for: DeepPheno: Predicting single gene loss-of-function phenotypes using an ontology-aware hierarchical classifier
Source: PLoS Comput Biol. 2020 Nov 18;16(11):e1008453. doi: 10.1371/journal.pcbi.1008453 (PMC7710064; doi:10.1371/journal.pcbi.1008453)
Supplement: Attachment — Submitted filename: response.pdf [file pcbi.1008453.s005.pdf]

Dear Dr Ma and Dr Greene,
Thank you for providing us with the opportunity to revise our manuscript. We would like to thank you and the reviewers for their comments which helped to improve our manuscript significantly. We have carefully revised our manuscript and have addressed all comments; we provide a point-by-point response below and you can find the modifications we made to our manuscript highlighted in red in our manuscript.
Please do not hesitate to contact us should you require any further information.
With best wishes, Maxat & Robert.

Editor:
Reviewer 3 notes certain structural issues with the manuscript, and I agree with these. I nearly sent the manuscript back before review, but elected in the end to send it out for review with plans to note the structural considerations at this stage.

Response: Thank you for the comment. We have now substantially restructured the manuscript, in particular the Methods, Results, and Discussion section, in response to this comment and the comments of Reviewer 3.

Editor:
As I was reading the manuscript, I also found myself thinking conceptually about the structure of the method proposed in Ma et al. (https://www.nature.com/articles/nmeth.46
Discussing how this work fits conceptually with related work in the area (also noted by Reviewer 3) would make it more accessible to the interested reader who may not know the details of every method within the field.

Response: Thank you for the comment. We have now added a comparison to DCell in the Discussion section. Briefly, DCell predicts (growth) phenotype from genotype, and uses the GO to design a deep neural network architecture; DeepPheno predicts (whole organism) phenotypes from function and uses the HPO structure to simulate (a kind of) physiology, i.e., the consequences of perturbing biological functions. From a technical perspective, DCell has a number of advantages over DeepPheno in that it explicitly represents biological entities as small neural network layers whereas DeepPheno uses the ontology structure only implictly; this allows DCell to be more interpretable than DeepPheno; however, there is a lot more training data available for DCell that allows it to make use of the larger number of parameters, while prediction of human phenotypes is mostly limited by the availability of data.

Reviewer #1: The authors are predicting genotype-phenotype association using a neural network where GO annotations of gene functions are used as features. The model is trained and tested on Human Phenotype Ontology (HPO) data and CAFA2 data. In terms of evaluation metrics, the authors are by and large using the metrics used in the CAFA2 project.
On the CAFA2 benchmark dataset DeepPheno has the highest $F_{\max}$ score compared to all the top methods in CAFA2. This shows the model is indeed learning to connect genotype with phenotype. On the CAFA2 training data sub-ontologies, the performance is mixed where in many cases other methods are performing better. Overall, the paper is increasing the state-of-the-art performance in many aspects for the genotype-phenotype association problem which is praiseworthy.
Having said that, I have the following comments about some aspects:
Reviewer 1:
1. I would like to compare the performance on CAFA2 datasets with a vanilla random forest that is taking the same input as DeepPheno as my first baseline. Random forests often surprisingly provide a strong baseline that is comparable or better than neural networks.

Response: We trained and evaluated RandomForest Regression model with all features that are used in DeepPheno. On the June 2019 dataset, the RandomForest model resulted in almost same performance in terms of Fmax and better performance in Smin evaluation. However, the neural network model was better in precision, AUPR and AUCROC evaluation metrics. On the CAFA2 dataset, RandomForest model also result in comparable performance, but the neural network model was slightly better in all evaluation metrics.
We have now included the comparison with the RandomForest baseline in the manuscript.

Reviewer 1:
2. I am not convinced about the novelty of hierarchical classifier the authors are claiming. I don't think this needs to be emphasized on the paper. Authors can provide their justification but this is a simple matrix multiplication.

Response: The novelty is that this operation is used to encode the ontology structure while training the model and contributes to the weight updates in the backprogation algorithm. We have now updated the introduction section and state more clearly the novelty of the algorithm.

Reviewer 1:

3. I am not convinced of the analysis of the false positive predictions the authors performed. I don't think the analysis done with the STRING dataset brings any confirmation or refutation of the false positives. The anecdotal examples mentioned in the False positive section also show that the majority of false positive predictions do not correspond to genotype-phenotype associations. The extrapolation by the authors seems a bit far fetched.

Response: We agree that the evidence we provided did not support the conclusion that we correctly predict genotype-phenotype relations. In response, we have added further analysis that shows that many false positive predictions in the dataset we used for our evaluation (released in June 2019) appeared in the dataset released in August 2020. We found that 898 specific (i.e., not propagated through the ontology) false positive predictions for 148 genes in the test set (20% of the June 2019 dataset) appeared in the August 2020 dataset. We also present two case studies that show these predictions and how DeepPheno was able to identify the phenotypes. We included this analysis in the manuscript.
Overall, the HPO database provides phenotypes for 4,366 genes; in the evaluation dataset, about 4 out of 5 human genes do not have annotations. Our STRING-based analysis is intended as a further indication (but not conclusive evidence) that some of our predictions for genes not yet in the HPO database may be correct.

Reviewer 1:
4. There is no guideline on the github page of the paper on how to apply their method on new datasets. Other researchers should be able to apply this method on their dataset with minimum knowledge.

Response: We have now published DeepPheno on the PyPI python package repository and updated the README with installation instructions; this will enable computational researchers to use our method and incorporate it in their own research.

Reviewer #2: Reproducibility Report has been uploaded as an attachment.

Response: We have now published deeppheno executable on PyPI and updated the README with installation instructions. We also fix the random seeds for splitting our datasets so that our simulations can be reproduced.

Reviewer #3: In this manuscript, the authors proposed a new gene-loss-of-function based phenotype prediction method that utilizes HPO terms, entitled DeepPheno, based on machine/deep learning. The study is relevant

as novel in silico approaches are always required in the fields of protein function prediction and disease association prediction. The manuscript is mostly written well in terms of the use of English; but the sectioning of the text is a little bit problematic. The proposed methodology is novel and effective; however, there are critical issues related to the current state of the manuscript. Below, I list my specific concerns/issues:

Major:

Reviewer 3:
1) The compartmentalization of the information into sections and sub-sections has issues, which makes it difficult to read the manuscript. For example, some technicalities regarding the structure of DeepPheno is provided at the beginning of the Results section, instead of the Methods section (i.e., the sub-section entitled DeepPheno Model should be under the methods). Additionally, some of the small-scale analyses are given in the discussion section (i.e., under False Positives sub-section), instead of the Results section. Sectioning should be re-considered from beginning to the end of the manuscript.

Response: We have significantly restructured our manuscript. All the results are now contained in the Results section, and we moved the description of the DeepPheno models to the Methods section; we replaced it with a single paragraph summarizing the model at the beginning of the Results.

Reviewer 3:
2) An additional test should be done considering the performance of DeepPheno on phenotypic terms from varying specificity groups. This grouping may either be based on the levels on the hierarchy of HPO or the number of annotated genes. Since DeepPheno uses deep neural networks and require a certain number of training instances to be able to predict a term, this analysis and its discussion may be critical and will help the reader in terms of machine learning algorithm selection according to the task and data at hand, in future predictive modeling studies.

Response: In addition to evaluation per each phenotype, we have added an evaluation based on branches of HPO in Supplementary Table 2. Also, we plotted the Fmax measure performance of each class by it specificity measure. The plot shows that there is no significant correlation between class specificity and prediction performance of the model.

Reviewer 3:
3) There should be a use-case study to indicate the real-world value of Deep-

Pheno, beyond performance calculations over the known data. This can be a case where DeepPheno predicts a specific gene-HPO association, which was not presented in the latest version of HPO, thus counted as a false positive. This case can be investigated over the literature to find an evidence for the predicted relation, as a means of validation. There already are a few examples in the discussion part regarding type II diabetes and GWAS; however, this example is also based on statistics and do not delve into the biological relevance of why those 4-5 genes should be associated with type II diabetes.

Response: Thank you for this comment, which we address by an additional analysis using a newer dataset (August 2020) of the HPO database. Our analysis shows that many false positive predictions in our dataset released in June 2019 appeared in the dataset released in August 2020. We found that 898 specific false positive annotations for 148 genes in the test set (20% of June 2019 dataset) appeared in the August 2020 dataset. We have now included this analysis in the manuscript. We further investigated two predictions in more detail, for phenotypes associated with NDUFA1 and GALC. NDUFA1 results in a metabolic disorder associated with NADH dehydrogenase deficiency, and we show how DeepPheno is able to correctly predict a range of phenotypes for this disorder from a set of functions; the predictions also give an explanation in terms of molecular pathways that result in the phenotypes observed in loss of function of NDUFA1. Similarly, we show the phenotype predictions for GALC and how they arise from the GO functions used as input to DeepPheno.

Reviewer 3:
4) There are two points regarding a related and cited study, HPO2GO. First of all, the idea of relating the occurrence of a phenotype to the lost function, and thus, using the GO annotations of genes/proteins to predict their phenotypic implications has already been proposed in the HPO2GO study. The same logic is used for DeepPheno as well. This inspiration should be mentioned in this manuscript by referring to HPO2GO in the relevant parts of the text, at least along with the other works that inspired this idea.

Response: We have included the HPO2GO study along with the methods which predict phenotypes associations from functions, and we emphasize the similarity of HPO2GO method with DeepPheno in the Introduction section.

Reviewer 3:
Second, considering Table 2 and Figure 2: In its own paper, HPO2GO's finalized CAFA2 performance is reported to be Fmax = 0.35. Here in thew

proposed manuscript, the reported performance of HPO2GO is similar to one of its sub-optimal versions explained in the corresponding article. Since CAFA challenge clearly states the train/test datasets to be used, and directly provides the performance calculation scripts, the results reported in different papers (where the authors state that they obey the CAFA rules) are directly comparable to each other. As a result, it is better to use the optimal CAFA2 results reported in the HPO2GO paper.

Response: We have now updated the Figure 2 and use the results reported in the HPO2GO paper and removed our evaluation results from Table 2.

Reviewer 3:
5) The information about the neural network layering is confusing. In figure 1 and the related text, it is stated that there are 2 layers: "The first layer is responsible for reducing the dimensionality of our sparse function annotation input and expression values are concatenated to it. The second layer is a multi-class multi-label classification layer with sigmoid activation functions for each neuron." However, in the Training and tuning of model parameters sub-section it is stated that the authors evaluated several models with two, three and four fully connected layer models. Where are these models and their performance results? What are the details of these 3-4 layered models? Did the authors settle for the 2 layered model at the end? If so, what was the reason behind? Was it that the 2 layered model performed the best? Or, did the performance gain by increasing the layers did not compensate for the increase in computational complexity?

Response: We train models with different sets up hyperparameters (including number of layers) while tuning model performance, and our best performing model has two layers. We select all hyper- parameters based on validation error. We describe these experiments now in the "Training and tuning of model parameters" subsection in the Methods.

Reviewer 3:
Related to this, a few hyper-parameters have been mentioned under Training and tuning of model parameters sub-section. How about the rest of the widely considered hyper-parameters in deep learning-based method development studies (e.g., using mini batches or not, if so, what is the size; and did you use batch normalization), have these been optimized as well, or the default value has been incorporated directly?

Response: We have now added this information to the subsection. We use

32 as size of the mini-batches and we did not use batch normalization layers. The remaining hyper-parameters were kept as default values set by tensorflow library.

Reviewer 3:
6) "Our phenotype prediction model is a fully-connected neural network followed by a novel hierarchical classification layer which encodes the ontology structure into the neural network. "
The technical details of this hierarchical classification layer are explained; however, this is a critical step in the proposed method, and the authors emphasize this as one of the important values added to the literature by this study. As a result, it should be explained and discussed in more detail. It is not clear how the hierarchical structure of the ontologies is taken into account inside the deep neural network. The authors stated that, this is achieved by multiplying the values of neurons at the second layer, with a matrix that represents the ontology structure, and then, max pooling is applied. Is this done to transfer, for example, the positive prediction given to a child term to its parent term, so that, even if the parent term did not receive a prediction itself, the system will change this to a positive prediction because its descendant received a positive prediction? If so, this will always behave towards increasing the number of predictions, and thus, the false positives. Moreover, are the flat versions the same models, only without this operation? Please provide precision and recall values in Table 1 along with the given metrics so that the reader can observe the effect of this operation and its advantages/disadvantages over the flat predictions. Please explain this hierarchical operation in more detail over a toy-example (e.g., imaginary prediction of an HPO term with and without the hierarchical processing).

Response: Yes, it is done to transfer prediction scores of child classes to parents when child classes have higher scores. In addition, this layer controls the flow of errors during the backpropagation process. We have now added a figure with a small example of the hierarchical classification function and also added precision and recall to Tables 1 and 2.

Reviewer 3:
7) The sizes of the feature vectors should be provided (in terms of both GO and gene expression).

Response: We have now included feature sizes in the Training and testing dataset section and updated the model architecture figure.

Reviewer 3:
8) “The best performance in AUROC of 0.75 among methods which use predicted phenotypes were achieved by our DeepPhenoAG and DeepPheno models. Table 4 summarizes the results. Overall, this evaluation shows that our model is predicting phenotype associations which can be used for predicting gene-disease associations.”
It is not clear how did authors draw this conclusion. Although higher than Naïve, AUROC values between 0.70-0.75 is not considered sufficiently high, so that they could be used with confidence. You can state that this is better compared to random prediction by a margin, but this value alone is not sufficient to say that DeepPheno can be used for predicting gene-disease associations, without further analysis.

Response: We agree with this comment and have now revised the sentence as suggested.

Reviewer 3:
9) “Specifically, it is designed to predict phenotypes which arise from a complete loss of function of a gene.”
Authors emphasize this at multiple locations of the manuscript; however, it’s not clear why this should be a complete loss of function. It can very well be a partial loss of function that give rise to the occurrence of a phenotype. Authors should either explain this in detail or change their statement.

Response: We agree with this comment and have now revised the statements throughout the manuscript. We also added further notes to the Discussion to address this point.

Minor: Reviewer 3:

1) “Similarity between observed phenotypes can be used to infer similarity between molecular mechanisms, even across different species. In humans, the Human Phenotype Ontology (HPO) [8] provides an ontology for characterizing phenotypes and a wide range of genotypephenotype associations have been created based on the human phenotype ontology.”
Please re-write these sentences, they are problematic in terms of the use of language.

Response: We have now revised the sentences as suggested.

Reviewer 3:
2) “We use 10% of the training set as a validation set to tune the parameters

of our prediction model. We generate 5 folds of random split and report 5-fold cross-validation results."
It is not clear how this is done. Normally, when a 5-fold CV is carried out it means that the training dataset is split into 5 pieces and each fold comprise 20% of the dataset. What is the meaning behind using 10% of the training set as a validation set here?

Response: We have many parameters to tune and we use early-stopping strategy to select the best model. This requires us to check the model performance every training epoch on a validation set. We cannot do it on the 20% test set because we have to keep it unseen during training phase. After we select our best model, we test and compute its performance on the 20% test set. We do this for all 5 folds and report average results.

Reviewer 3:
3) "We use interactions with a score of 700 or above in order to filter high confidence interactions."
Scores in StringDB vary between 0 and 1, should this be 0.7?

Response:
Yes, we have now updated the manuscript accordingly.

Reviewer 3:
4) "The value of the vector si at position j is 1 iff pj is a (reflexive) ..."
Typo.

Response: Fixed.
